# New Short Tandem Repeat-Based Molecular Typing Method for *Pneumocystis jirovecii* Reveals Intrahospital Transmission between Patients from Different Wards

**DOI:** 10.1371/journal.pone.0125763

**Published:** 2015-05-01

**Authors:** Maud Gits-Muselli, Marie-Noelle Peraldi, Nathalie de Castro, Véronique Delcey, Jean Menotti, Nicolas Guigue, Samia Hamane, Emmanuel Raffoux, Anne Bergeron, Sandrine Valade, Jean-Michel Molina, Stéphane Bretagne, Alexandre Alanio

**Affiliations:** 1 Laboratoire de Parasitologie-Mycologie, AP-HP, Groupe Hospitalier Saint-Louis-Lariboisière-Fernand-Widal, Paris, France; 2 Service de transplantation rénale, AP-HP, Groupe Hospitalier Saint-Louis-Lariboisière-Fernand-Widal, Paris, France; 3 Université Paris-Diderot, Sorbonne Cité, Paris, France; 4 Service de Maladie Infectieuses et tropicales, AP-HP, Groupe Hospitalier Saint-Louis-Lariboisière-Fernand-Widal, Paris, France; 5 Service de Médecine interne, AP-HP, Groupe Hospitalier Saint-Louis-Lariboisière-Fernand-Widal, Hôpital Lariboisière, Paris, France; 6 Institut Pasteur, Unité de Mycologie Moléculaire, Centre National de Référence Mycoses invasives et Antifongiques, Paris, France; 7 CNRS URA3012, Paris, France; 8 Service d’Hématologie adulte, AP-HP, Groupe Hospitalier Saint-Louis-Lariboisière-Fernand-Widal, Paris, France; 9 Service de Pneumologie, AP-HP, Groupe Hospitalier Saint-Louis-Lariboisière-Fernand-Widal, Paris, France; 10 Service de Réanimation, AP-HP, Groupe Hospitalier Saint-Louis-Lariboisière-Fernand-Widal, Paris, France; University Hospital San Giovanni Battista di Torino, ITALY

## Abstract

*Pneumocystis* pneumonia is a severe opportunistic infection in immunocompromised patients caused by the unusual fungus *Pneumocystis jirovecii*. Transmission is airborne, with both immunocompromised and immunocompetent individuals acting as a reservoir for the fungus. Numerous reports of outbreaks in renal transplant units demonstrate the need for valid genotyping methods to detect transmission of a given genotype. Here, we developed a short tandem repeat (STR)-based molecular typing method for *P*. *jirovecii*. We analyzed the *P*. *jirovecii* genome and selected six genomic STR markers located on different contigs of the genome. We then tested these markers in 106 *P*. *jirovecii* PCR-positive respiratory samples collected between October 2010 and November 2013 from 91 patients with various underlying medical conditions. Unique (one allele per marker) and multiple (more than one allele per marker) genotypes were observed in 34 (32%) and 72 (68%) samples, respectively. A genotype could be assigned to 55 samples (54 patients) and 61 different genotypes were identified in total with a discriminatory power of 0.992. Analysis of the allelic distribution of the six markers and minimum spanning tree analysis of the 61 genotypes identified a specific genotype (Gt21) in our hospital, which may have been transmitted between 10 patients including six renal transplant recipients. Our STR-based molecular typing method is a quick, cheap and reliable approach to genotype *Pneumocystis jirovecii* in hospital settings and is sensitive enough to detect minor genotypes, thus enabling the study of the transmission and pathophysiology of *Pneumocystis* pneumonia.

## Introduction

*Pneumocystis jirovecii* is an unusual ubiquitous fungus that specifically infects humans [1,2]. This fungus causes *Pneumocystis* pneumonia (PCP) in T cell-deficient patients, including HIV-positive, solid organ transplant and cancer patients, but also in adults and children with various underlying immunological diseases [3–9]. Primary infection occurs in early childhood with about 85% of children exposed in the first 18 months of life, as revealed by the detection antibodies against *P*. *jirovecii* or fungal DNA [1,2]. Immunocompetent hosts are thought to act as a reservoir for *P*. *jirovecii* and may expose immunocompromised individuals via airborne transmission [10–12]. This notion has been confirmed epidemiologically by numerous PCP outbreaks resulting from intra-hospital transmission, especially in renal transplant units [13–22]. From a pathophysiological perspective, understanding transmission will help us to determine whether: (i) *P*. *jirovecii* remains dormant after the control of a primary infection in childhood and reactivates upon immunodeficiency [2,23]; or (ii) individuals are continuously exposed to *P*. *jirovecii* throughout life and achieve balance between clearance and exposure until the latest *P*. *jirovecii* genotype(s) leads to the development of PCP [1,2]. However, these two hypotheses are not necessarily mutually exclusive. Furthermore, knowledge of *P*. *jirovecii* transmission in the hospital setting is important to adapt preventive measures.

*Pneumocystis jirovecii* thrives at the surface of alveolar type I pneumocytes and was only recently amplified *in vitro* for the first time on an air-liquid interface culture system [24]. Until now, the uncultivable feature of *Pneumocystis* makes more difficult the studies on the genetic diversity and evolution of the pathogen in humans. Since the 1990’s, different tools were developed to study genetic diversity, each with their own advantages and disadvantages. In particular, the sensitivity of the methods to multiple genotypes detection in a given sample differs. Single-strand conformation polymorphism (SSCP) was initially used [25–27], but was replaced with multilocus sequence typing (MLST) which involves direct DNA sequencing. Recently, a MLST scheme based on single round PCR of three to eight partial gene sequences, provided a Simpson’s index of diversity of 0.987 and 0.996, respectively [28]. However, technologies based on Sanger sequencing can detect mixtures with a ratio between 1:3 and 1:10 [29]. For this reason, single base extension technology based on the detection of specific SNPs was developed [30], and detects mixtures with a higher sensitivity than technologies based on Sanger sequencing [30]. Recent studies have also proposed the analysis of microsatellite markers, also called short tandem repeat (STR) analysis, to genotype various fungi [31–35]. This technique is simple, cheap, reproducible, can be standardized [36,37] and can easily detect multiple genotypes in samples [31,38]. Here, we developed a new panel of STR markers to obtain a simple, rapid and reliable tool for investigating *P*. *jirovecii* transmission in hospital settings. We tested this method on a panel of samples collected in our hospital.

## Material and Methods

### Identification of short tandem repeat loci

Short tandem repeats (STRs) were identified from the recently published *P*. *jirovecii* nuclear genome [39]. The 358 contigs of the genome were screened for STRs using Tandem repeat Finder software (http://tandem.bu.edu/trf/trf.html) [40]. A minimum alignment score of 50 and a maximum period size (repeat unit) of 5 nucleotides was used, and gave 179 putative results. Di- and tri-nucleotide repeats based on loci with the highest repeat numbers were then selected and loci containing mixed or partial repeat sequences were rejected.

The 10 markers with the most repeat units, distributed in different regions of the genome (different contigs) and in different locations relative to the coding sequence were selected *in silico*. These regions were then amplified with primers in the 3’ and 5’ flanking regions of the repeats that were designed in Primer 3 software (http://primer3.ut.ee), with the aim of obtaining amplicons of various sizes for each marker with no overlap between markers. An initial test on 10 randomly selected DNA samples (10 patients) showed correct amplification for six of the 10 selected markers in all samples. These six markers were retained for further investigation (Table 1). Four of these markers were tri-nucleotide repeats, located in the contigs 022 (STR*Pj*_3_022), 108 (STR*Pj*_3_108), 138 (STR*Pj* _3_138) and 279 (STR*Pj* _3_279), and the other two were di-nucleotide repeats, located in contigs 189 (STR*Pj*_2_189) and 278 (STR*Pj*_2_278). Two were intronic (located in an intron; STR*Pj*_3_022 and _279), two were exonic (located in an exon, STR*Pj*_3_108 and _138) and two were extra-genic (located in the 5’ or 3’ flanking region of a gene, STR*Pj*_2_189 and _278) (Table 1).

**Table 1:** Characteristics of the STR markers and primers used in this study.

Primer name	Repeatunit	*P*. *jirovecii* genomic location (contig)	*P*. *murina* corresponding chromosome	Forward and reverse primer sequence	Amplicon size (bp)	Numbers of observed alleles
**STR*Pj*_3_022**	3	Intronic (022)	13	F: TTGGCAATGAATCAATAATCGT	140	3
				R: TCTGAGTAAAAGATGGTGAAAGA		
**STR*Pj*_3_108**	3	Exonic (108)	08	F: TGCCTCAATATCATCAATGTCA	141	4
				R: AGAGAAGATCAAGGAGAGGA		
**STR*Pj*_3_138**	3	Exonic (138)	07	F: TCCACGAACTAGCTTGTTAGT	170	10
				R: TAGAGCATTCGGTTCCTACT		
**STR*Pj*_2_189**	2	Extra-genic (189)	04	F: CTCGAAAACGGTTTCTAGATCA	205	24
				R: GTCCAGAAAATAAGATCATGCTGA		
**STR*Pj*_2_278**	2	Extra-genic (278)	16	F: ATCAGCAAACTCCTCAGGAT	187	5
				R: AGGTTTTGGACGTTTGAAAA		
**STR*Pj*_3_279**	3	Intronic (279)	06	F: GGACGATATTGATAATCTGTTAGCT	180	6
				R: GGTCTGTCATTAAACAAGCCA		

The 2500 bp sequence up and downstream from each marker was aligned to the *Pneumocystis murina* genome (available at http://www.broadinstitute.org/annotation/genome/*Pneumocystis*_group.2/MultiHome.html). The markers were broadly distributed in the genome and were probably located on different *P*. *jirovecii* chromosomes (Table 1). Indeed, since *P*. *jirovecii* contigs are still not assembled as chromosomes, as opposed to *P*. *murina* genome, the precise location of these markers in *P*. *jirovecii* genome is still uncertain.

### PCR amplification and genotyping

The six selected STRs were amplified separately by PCR since multiplexing failed to be as sensitive as single PCR (data not shown). The forward primers were tagged with fluorophores (FAM, HEX or ATTO565). All PCR reactions were performed on a GeneAmp PCR System 9700 Thermocycler (Applied Biosystems) in a final volume of 20 μL containing 1X Ampli Taq Gold buffer (Life technologies) with 0.25μM of each primer, 2.5mM of MgCl2, 0.8μM of dNTPs, 0.25 UI of Ampli Taq Gold polymerase (Life technologies) and 2 μL of DNA. The reaction consisted of 10 minutes at 95°, followed by 35 cycles of 30 s at 95°C (denaturation), 30 s at 56° (primer annealing) and 60 s at 72°C (extension) followed by a final extension of 10 min at 72°C. A sample with a mixed genotype at one locus (sample181) was used in each PCR run as an internal control and to measure reproducibility.

### Fragment processing and analysis

After amplification, 2 μL of PCR product was prepared for fragment analysis by the addition of 18 μL of formamide (3700 formamide, Life technologies) and 1 μL of Genescan-500 LYZ Size Standard (Life technologies). Capillary electrophoresis was performed with the denaturing polymer POP-7 (Life technologies) in a 50 mm capillary tube at 60°C. The lengths of the PCR fragments were determined on an ABI 3500 genetic analyzer with ABI Gene mapper v4.1 software (Life technologies).

### Minority allele detection

To test the limit of detection of multiple genotypes, two samples were selected containing both a single allele/locus and the same fungal load (same Cq +/- 1), which gave rise to a peak intensity in a 1:1 ratio. Various serial dilutions (1:1000, 1:100; 1:50, 1:20, 1:10 and 1:2) of each DNA sample were then tested, and the presence of the expected amplicons was analyzed in each mixture.

### Genotyping

Genotypes were determined if (i) each of the six markers were pure (no additional peak corresponding to a smaller or larger amplicon); or (ii) only one marker had multiple amplicons (one to three additional detectable peaks corresponding to a smaller or larger amplicon). In samples harboring mixtures in more than one marker, already known genotypes in other types of samples (see above) (n = 61) were screened. Deduced genotypes that were detected only in those samples were considered not suitable for global analysis. In samples harboring several alleles at one locus, the major allele was recorded (entered first in Tables).

### Samples and patients

A total of 106 respiratory samples defined as positive for *P*. *jirovecii* by quantitative real-time PCR [41] and harboring a high fungal load (mean quantification cycle = 25.46±4.25) were selected from those collected between 1^st^ January 2011 and 31^st^ December 2013 [41]. DNA was extracted as described previously [41] and stored at -20°C. All samples were processed for diagnostic procedures with the patient’s informed consent. The patients were cared for in three hospitals in the north of Paris. Demographic data and clinical variables including age, sex, underlying disease at the time of the BAL procedure and outcome at the last follow-up visit were recorded retrospectively from the electronic medical file. Underlying diseases were divided into four categories (HIV positivity, hematological malignancies, solid organ transplantation, others). Each hospital stay, as an inpatient or an outpatient, in addition to radiological and medical consultations, was recorded with electronic medical file software.

### Ethics statement

This study was a non-interventional study with no change in the usual procedures. Biological material and clinical data were obtained only for standard diagnostic following physicians’ prescriptions with no specific sampling. According to the French Health Public Law (CSP Art L1121-1.1), such protocol does not require approval of an ethics committee and is exempted from specific informed consent application. However, all patients were informed and signed documents for non-opposition to this study. Data file was declared and approved to the French data protection agency (n°1818924).

### Data analysis

Relatedness between the different genotypes was investigated by comparing allelic profiles with the minimum spanning tree (MStree) method (BioNumerics software v6.5, Applied Maths Inc., Austin, TX). Briefly, STRs were treated as multistate categories based on an infinite allele model (i.e., all changes are equally likely). Singletons were defined as genotypes that were not grouped into clonal complexes, i.e. they had at least two allelic mismatches with any other genotype. The number of repeat differences between ancestral and derived alleles was computed for each link of one mismatch along the MStree. The classical criterion of one allelic mismatch to group genotypes in clonal complexes was used [42].

Discriminatory power was calculated with Simpson’s diversity index (*D*) as described previously [31,43], taking into account only one sample per genotype per patient (samples harboring a genotype already recovered once in a given patient were excluded) such that only independent samples were retained [43].

Correlation with clinical data was performed only with one sample per patient. Statistical analysis was performed with Prism v5.0 (GraphPAD Software, San Diego, CA).

## Results

### STR-based method

**Fig 1:**
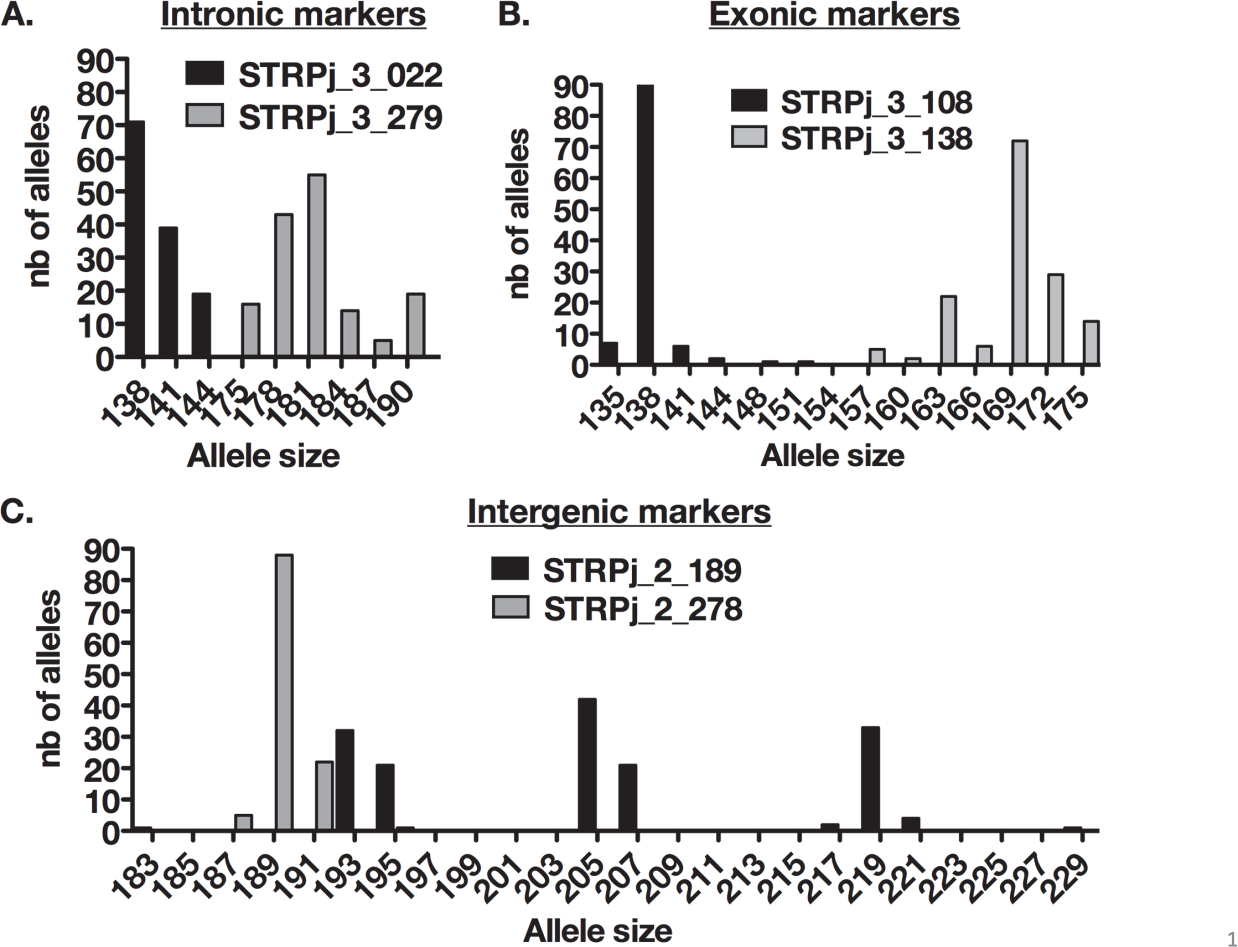
Distribution of the different alleles in the 106 samples tested for the intronic (A), exonic (B) and intergenic (C) markers.

A total of 106 *P*. *jirovecii*-PCR-positive samples from 91 patients were genotyped at our six loci. The allelic diversity varied in our population (Fig 1) with STR*Pj*_3_022, STR*Pj*_3_108, and STR*Pj*_2_278 harboring a low and STR*Pj*_3_138, STR*Pj*_2_189, STR*Pj*_3_279 a high allele diversity in size (Fig 1, Table 1). We tested the ability of our assay to detect multiple alleles for each marker in one sample. The maximal ratio allowing the detection of the minor allele was 1:50 (2%) (data not shown). In the internal control sample tested nine times in nine PCR runs, a variation in the percentage of the minority allele compared to the major allele (2.3 to 9.4%) was observed (S1 File).

Samples could be divided into three categories: (i) 34 (32.1%) samples and 32 (35.2%) patients had a unique genotype, i.e. one allele detected at each locus (Table 2 and S2 File); (ii) 24 (22.6%) samples and 23 (25.2%) patients had a multiple genotype with more than one allele detected at one locus (Table 2 and S2 File); and (iii) 48 (45.3%) samples and 41 (45.1%) patients had a multiple genotype with more than one allele at two to six loci (Table 2 and S3 File)). Multiple genotypes were detected in 72 (67.9%) samples and 61 (67%) patients.

**Table 2:** Summary of genotype categories for *P*. *jirovecii* samples.

	No. of samples (%) n = 106	No. of patients (%) n = 91
**Single genotype**	34 (32.1)	32 (35.2)
**Multiple genotypes**	72 (67.9)	61 (67)
** Multiple genotype in 1 marker**	24 (33.3)	20 (32.8)
** Multiple genotype in >1 marker**	48 (66.7)	41 (67.2)
** Multiple genotype in 2 markers**	21 (43.7)	18 (43.9)
** Multiple genotype in 3 markers**	14 (29.2)	11 (26.8)
** Multiple genotype in 4 markers**	11 (22.9)	10 (24.4)
** Multiple genotype in 5 markers**	1 (2.1)	1 (2.4)
** Multiple genotype in 6 markers**	1 (2.1)	1 (2.4)

The *P*. *jirovecii* fungal load determined from the quantification cycle (Cq) using mtLSU diagnostic qPCR was not significantly different in unique and multiple genotype samples (*p* = 0.45), suggesting that the ability to detect mixtures was not dependent on fungal load.

### Analysis of iterative samples in patients

Thirty respiratory repeat samples (BAL and/or induced sputum) from 15 patients (two per patient) were available. These samples were taken at intervals ranging from 0 to 37 days (Table 3). At least one allele was shared by the two samples in all patients and for all markers except in two patients, who had one marker that showed a one repeat contraction or amplification between samples (Patient 21, marker#189; Patient 23, marker#278).

**Table 3:** Short tandem repeat typing results for 30 iterative samples from 15 patients (two per patient).

No. of patients	Background of the patient	No. of Induced samples	Type of sample	Interval between first and second sample (days)	Cq	Induced sTR*Pj* amplicon size (bp)					
						**#022**	**#108**	**#138**	**#189**	**#278**	**#279**
**07**	Hematology	109***	Induced sputum		20.7	141/144	138	169/175	195/219	189	181/190
		110***	Induced sputum	+1	21.6	141/144	138	175/169	195/219	189	181/190
**10**	Others	240	Induced sputum		27.2	144	138	169	219/207*	189	190/175*/178*
		243	Induced sputum	+7	26.8	144	138	169/175*	219	189	190
**12**	Hematology	197	Induced sputum		28.2	138	138	172	195	189	181
		199	BAL	+1	22.6	138	138	172	195	189	181
**13**	Others	079	BAL		31.3	138/141*	138	169	221/195	189	181/187
		080	Induced sputum	0	30.0	138	138	169	221/195	189	181/187
**14**	HIV-positive	319	Induced sputum		20.6	141/138	138	172	219	189/191	181/190
		320	Induced sputum	0	23.0	141/138	138	172	219	189/191	181/190
**15**	Others	045***	BAL		20.4	138	138*/144	169/163**	193/207**	189	175
		053***	BAL	+14	28.6	138	144	169/151**	193/195**	189	175
**16**	HIV-positive	311	Induced sputum		23.2	138	138	163/169	205/193	189	175/178
		316	BAL	+14	22.0	138	138	163/169	205/193	189	175/178
**17**	Renal transplant	165	Induced sputum		23.5	144	138	169	219	189	190
		168	BAL	+1	28.9	144	138	169/166*	219	189	190
**18**	HIV-positive	272	Induced sputum		16.8	138/141*	138	169/166*	219/205/193	191	181
		282	BAL	+18	26.7	138	138	169	219/205/193	191	181
**19**	HIV-positive	249	Induced sputum		26.0	138	138	172	195	189	181
		252	BAL	+1	17.8	138	138	172	195	189	181
**20**	HIV-positive	030	Induced sputum		23.1	138	138	169	205/195	189	181
		032	BAL	+2	19.2	138	138	169	205/195	189	181
**21**	HIV-positive	132***	Induced sputum		28.2	138/141*	138	169/163	207**	189/191	178/181*/184
		134***	Induced sputum	+1	26.4	138	138	169/163	205**	191/189	184/178
**22**	HIV-positive	309***	Induced sputum		20.1	141	138	169/175	183**/207	189	178
		321***	BAL	+37	28.1	141	138	169/175	207/205**	189	178
**23**	HIV-positive	212***	Induced sputum		28.9	138/144*	138	157*/169	205	189**	181
		215***	BAL	+3	29.1	138	138	169	205	191**	181
**25**	HIV-positive	158	Induced sputum		21.2	138	138	169/163*	205	189	178/181/184
		160	Induced sputum	+1	27.4	138	138	169	205	189	178/181/184

When multiple alleles were detected, the major allele is written first.

* gain or loss of allele between the two samples.

** allele replacement between the two samples.

*** samples in which the ratio of a given marker differed between the first and second sample.

An identical composition (including unique and mixed genotypes) was observed for 6/15 (40%) patients (Patient12, 14, 16, 19, 20, 24) in samples taken up to 14 days apart. A gain or loss of alleles was observed in 8/15 (53%) patients (Patient13, 15, 17, 18, 21, 23, 25, 26) at various loci, and allele replacement occurred in 4/15 (26.7%) patients (Patient15, 21, 22, 23) in samples taken up to 37 days apart (Table 3).

Change in the allele ratio was observed in five patients (Patient 07, 15, 21, 22, 23) harboring multiple genotypes (Table 3). These samples were obtained between 1 (Patient07, Patient21) and 37 days apart.

### Correlation with patient data

Among the 91 patients, 41 (45.1%) were HIV-positive, 25 (27.5%) had hematological malignancies, 14 (15.4%) were renal transplant recipients, and 11 (12.1%) had other immunodeficiencies (solid tumors, immunosuppressive therapy or constitutive immune defects). All patients were cared for in three healthcare facilities: Saint-Louis (n = 77, 84.6%), Lariboisière (n = 11, 12.1%), and Robert Debré (n = 3, 3.3%) hospitals. The male:female ratio was 2.3 and median age was 52 years old [range 1–84]. Samples with multiple alleles at several loci were excluded from genotyping to avoid misassignment of a combination of alleles to a given genotype leading to artifactual diversity. A total of 61 genotypes were detected in 55 (51.9%) samples (54 [59.3%] patients) (S2 File).

We analyzed the distribution of each allele in the six markers for each disease groups taking into account every allele detected in all samples (unique or multiple alleles for each marker) and the proportion of each allele for each marker in each disease group. The distribution of marker#022, #108 (*p* = 0.002), #138 (*p* = 0.040), #189 (*p* = 0.001), and #279 (*p*<0.0001) significantly differed between groups (S1 Table). Allele 144 in marker#022 (*p*<0.0001), allele 138 in marker#108 (*p* = 0.049), allele 169 in marker#138 (*p* = 0.015), allele 219 in marker#189 (*p*<0.0001) and allele 190 in marker#279 *p*<0.0001) were more frequently observed in renal transplant patients than in other patients. Of note, allele 144 in marker#022 was less frequently observed in HIV patients (*p* = 0.0003) than in other patients. Kidney transplant patients tended to harbor more samples with a unique genotype than other patients (8/17 [47%] vs. 22/77 [29%], respectively; *p* = 0.061).

**Fig 2:**
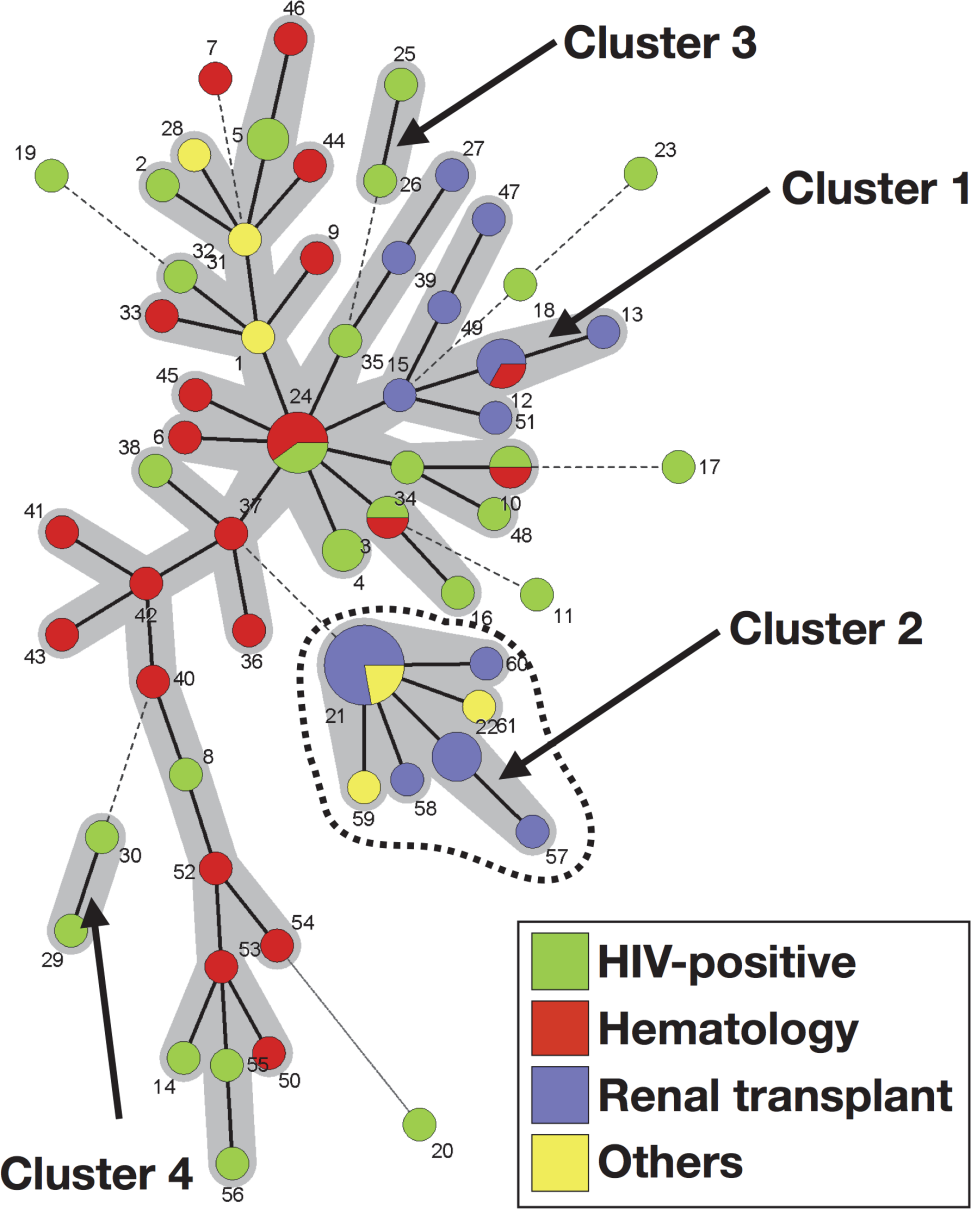
Minimum spanning tree analysis of 61 genotypes from 55 samples harboring a unique genotype (one allele per marker) or multiple genotypes (multiple alleles in one marker). The number of allelic mismatches among STR profiles was used as distance. Each circle corresponds to one genotype (Gt), with its arbitrary number indicated next to it. The size of the circle is correlated with the number of isolates possessing the corresponding Gt, from one (smallest circle) to nine (Gt21). Dark, dashed and thin connecting bars corresponds to one, 2 or >2 different markers observed between linked genotypes. Gray zones surrounding some groups of circles indicate that these profiles belong to the same genetic cluster, meaning that they have a single allelic mismatch with at least one other member of the group. Cluster 2, which was significantly associated with renal transplant recipients, is shown by a dashed line. The color of the circles indicates the underlying disease of the patient in whom this specific genotype was recovered (Green, HIV patient; Red, hematology patient; Purple, renal transplant recipient; Yellow, other cause of immunosuppression).

Minimum spanning tree (MSTree) analysis of 55 samples (54 patients) revealed four clusters, and seven singletons corresponding to seven samples (seven patients) (Fig 2). Cluster 1 was composed of 43 genotypes (34 samples [34 patients]), cluster 2 was composed of seven genotypes (12 samples [11 patients]), and clusters 3 and 4 were composed of two genotypes (one sample [1 patient], each) (Fig 2). Allele 144 in marker#022 (*p*<0.0001), allele 169 in marker#138 (*p* = 0.014), allele 219 in marker#189 (*p*<0.0001), and allele 190 in marker#279 (*p*<0.0001) were more frequently associated with cluster 2 than the other genotype categories.

Sex and hospital did not cluster with the five genotype categories obtained in the MSTree (clusters 1, 2, 3, 4, and the singletons) (data not shown). However, underlying diseases were not evenly distributed in the five genotype categories (*p* = 0.0008). A higher proportion of samples from kidney transplant recipients (9/11 [81.8%]) was observed in cluster 2 than in the other categories (6/44 [13.6%]) (*p*<0.0001). A higher proportion of samples from HIV-positive (6/7 [85.7%]) patients was observed in the singletons than in the other categories (15/48 [31.2%]) (*p* = 0.0097).

Genotype 21 (Gt21), composed of alleles 144, 138, 169, 219, 189, 190 in marker#022, #108, #138, #189, #278 and #279, respectively, was found in 8/54 (14.8%) patients of this dataset. In addition, the corresponding alleles were found in three samples (two patients) harboring multiple genotypes, suggesting transmission to these patients (Patients07 and 09). We investigated these 10 patients (six renal transplant recipients, two hematological malignancies and two others) to search for potential transmission events and recorded their presence in the hospital before and at the time of PCP diagnosis (Table 4).

**Table 4:** Characteristics of the 10 patients in whom genotype 21 was detected.

No. of patient	Sex	Hospital	Geographical origin	Background	Sample ID	Sample type	Ct	STRs markers						Gt
								**#022**	**#108**	**#138**	**#189**	**#278**	**#279**	
**1**	M	Hospital 1	France	Renal transplant	26	BAL	20.7	**144**	**138**	**169**	**219**	**189**	**190**	**21**
**2**	M	Hospital 1	Caribbean islands	Renal transplant	37	BAL	23.9	**144**	**138**	**169**	**219**	**189**	**190**	**21**
**3**	M	Hospital 1	Ivory coast	Other (cancer)	28	IS	30.2	**144**	**138**	**169**	**219**	**189**	**190**	**21**
								144	138	163	219	189	190	59
**4**	F	Hospital 1	France	Renal transplant	219	IS	21.5	**144**	**138**	**169**	**219**	**189**	**190**	**21**
**5**	M	Hospital 1	France	Renal transplant	165	IS	23.5	**144**	**138**	**169**	**219**	**189**	**190**	**21**
					168	BAL	28.9	**144**	**138**	**169**	**219**	**189**	**190**	**21**
								144	138	166	219	189	190	60
**6**	M	Hospital 1	France	Renal transplant	196	BAL	23.0	**144**	**138**	**169**	**219**	**189**	**190**	**21**
**7**	M	Hospital 1	Africa	Hematology	109	IS	20.7	141/**144**	**138**	175/**169**	195/**219**	**189**	181/**190**	21*
					110	IS	21.6	141/**144**	**138**	175/**169**	195/**219**	**189**	181/**190**	21*
**8**	M	Hospital 1	France	Renal transplant	181	BAL	14.6	**144**	**138**	**169**	**219**	**189**	**190**	**21**
								144	135	169	219	189	190	58
**9**	M	Hospital 1	France	Hematology	231	BAL	28.3	**144**/138	**138**	**169**/157	**219**/193	**189**/191	**190**/181/178	21**
**10**	M	Hospital 1	na	Other (cancer)	240	IS	27.17	**144**	**138**	**169**	**219**/207	**189**	**190**/175/178	21**
					243	IS	26.8	**144**	**138**	**169**	**219**	**189**	**190**	**21**
								144	138	175	219	189	190	61

The major allele is written first for samples with multiple alleles. Bold numbers show alleles from Gt21.

21*, 21** are samples harboring multiple genotypes in which all alleles from Gt21 were present in addition to other deduced genotypes. In samples 109 and 110 (21*), alleles corresponding to Gt21, were minority alleles (lower intensity of the peak of the G21 allele compared to the peak of the other allele) in all mixed markers. In samples 231 and 240 (21**), alleles corresponding to Gt21 were major alleles (higher intensity of the peak of the Gt21 allele compared to the peak of the other allele).

**Fig 3:**
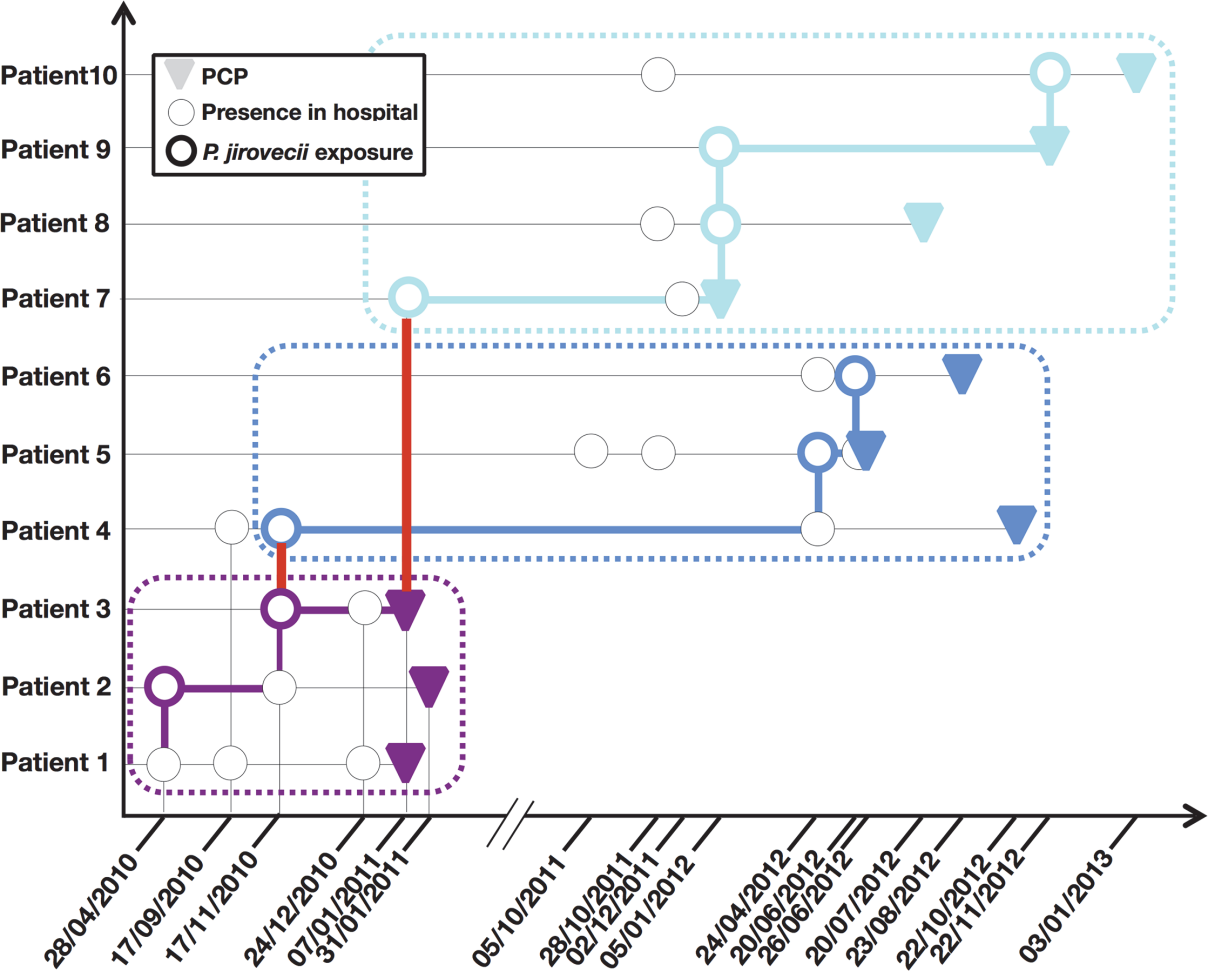
Transmission map of the 10 patients in whom the *P*. *jirovecii* genotype 21 was detected. The date corresponds to the time at which the patient was present in the hospital and is delineated as a circle. Colored circles show *P*. *jirovecii* exposure of a given patient on a given day. Color coding distinguishes the different groups of patients (purple for patients 01–03, blue for patients 04–06 and turquoise for patients 07–10). The red bar corresponds to transmission between two groups of patients. Colored bars show the transmission routes.

We discovered several instances where patients could have met, potentially leading to transmission. In particular, three groups of patients were temporally linked (Patients01-03, Patients04-06 and Patients07-10). Many patients were present in the same place at several instances before and during the PCP episode (Fig 3). Patient01 was the index case that initiated the transmission chain. Patient01 and 03 had the first recorded cases of PCP among these 10 patients. Patient03 also potentially played a central role in the transmission, with links to Patients04 and Patient07 (red bars). Patient04 was a long-term carrier (705 days between exposure and PCP) with putative transmission to Patient05. The median time between putative exposure and PCP was 197 days (range: 42–705 days).

In the 48 samples with two or three alleles at two to six loci, at least one of the already determined 61 genotypes were searched for and were found in 40 (83%) samples. A median of 2 genotypes/sample (range [1–10]) was found. Genotype 3 (Gt3) and Genotype 1 (Gt1) were found in 9/48 and 8/48 samples, respectively (S2 and S3 files). However, no epidemiological link was found in the samples harboring Gt1 or Gt3.

### Discriminatory power of the assay

Simpson’s index of diversity (D) was calculated from the three markers harboring the most allelic diversity in the entire set of six markers. Given the potential transmission of Gt21 in cluster 2, calculations were performed with the whole data set but also after the removal of potentially linked cases, i.e. cluster 2 (43 patients) and renal transplant samples from cluster 2 (45 patients) (Table 5). Six markers gave a better D index than 3 to 5 markers (Table 5). The D index was 0.985 for the six markers calculated from the whole population (54 patients) (Table 5). A higher D index (D = 0.992) was obtained with the 6 markers when epidemiologically linked samples were excluded (Table 5).

**Table 5:** Discriminatory power of the assay for genotyping *P*. *jirovecii*.

Name of markers included	No. of markers included	Simpson’s index of diversity (D)		
		Whole data (n = 54 patients)	wo cluster 2 Gt (n = 45 patients)	wo renal transplant of cluster 2 (n = 45 patients)
**STRPj_138/189/279**	3	0.949	0.957	0.964
**STRPj_022/138/189/279**	4	0.963	0.981	0.983
**STRPj_022/138/189/278/279**	5	0.980	0.989	0.990
**STRPj_022/108/138/189/278/279**	**6**	**0.985**	**0.992**	**0.992**

## Discussion

Here, we described a short tandem repeat (STR)-based genotyping assay including six markers. One marker (marker STR*Pj_*2_278) has already been used (PjMS5) in a recent publication [44]. The six markers are located in several contigs within genes (exonic, intronic) or at intergenic locations. Alignment to the *P*. *murina* genome suggests that our markers are located on six different chromosomes (Table 1). Exonic and intronic markers are expected to have lower allelic variability than intergenic markers because these regions are under constraint [45]. However, in our case, each marker harbored allelic variability regardless of its location with respect to a gene. The assay is convenient and is based on a single PCR run. It also has high discriminatory power and enables the detection of multiple genotypes with a maximal ratio of 1:50 (2%), which is more sensitive than Sanger sequencing [29] and SSCP [46].

We established the stability of our six markers by analyzing samples taken from the same patient: in 13/15 patients, the same alleles was iteratively found in 6/6 markers in samples taken between 0 and 14 days apart and in all 15 patients, in 5/6 markers in samples taken between 0 and 37 days apart. The analysis revealed the occurrence of allele replacement or modification (contraction or extension of 1 to 6 repeats for a given marker at 1 or 2 loci) for 4/15 patients, suggesting the microevolution of *P*. *jirovecii* during infection (over a short period of time, up to 37 days). This reveals rapid variation of these parts of the genome due to their repetitive nature. Indeed, infection is associated with fungal proliferation and modification, and the number of repeat units could be easily altered (gain or loss). Unfortunately, the stability of each selected marker, as was reported for STR*Af* in *Aspergillus fumigatus* [31], cannot be assessed experimentally in humans during infection nor *in vitro* with long-term cultures of *P*. *jirovecii*. However, the culture system published recently [24] or murine model of *Pneumocystis* pneumonia with *P*. *murina* could be used to test the stability of the markers and to study the plasticity of *P*. *jirovecii* nuclear and mitochondrial genomes. Comparison of STR to sequence-based typing methods will also bring new arguments to assess the level of stability of these STR markers.

In our study, about 70% samples harbored multiple genotypes. Sanger sequencing-based methods are less sensitive and detected multiple genotypes in only about 30% of samples [28,47]. A high number of multiple genotypes were also observed with SSCP studies in HIV patients [48] or with an STR-based method (70% of patients) [44], suggesting that different strains of *P*. *jirovecii* are constantly transmitted between humans throughout life [1,2]. Recovery of unique versus multiple genotypes was not significantly associated with underlying disease, although samples from renal transplant patients tended to harbor a unique genotype, thus reinforcing the hypothesis of transmission between these patients, as already described in literature [18,19,22].

The ability to detect multiple genotypes makes the data difficult to interpret but enables the identification of minor alleles. Association of major and minor allele has been used to determine genotypes using microsatellite [44] and SSCP [46] typing methods.

From a technical point of view, we think that PCR amplification of STR markers could introduce variation in the allele ratio in mixed samples. Indeed, a variation (2.3 to 9.4%) was observed in the proportion of the minority allele compared to the major allele. Consequently, it is difficult to be confident in the results when determining the different genotypes in complex allele mixtures (multiple alleles at more than one marker) including various minority alleles, unless one knows precisely what to look for, as was the case for Gt21. We searched for each of the 61 genotypes determined in pure or mixed samples at one locus in mixed samples two or three alleles at two to six loci. The Gt21 alleles were observed in four samples containing multiple genotypes, thus demonstrating potential transmission because the patients formed part of a transmission route. In our case, Gt21 alleles represent either the minority allele in all markers (sample109 and 110) or the majority allele in all markers (sample231 and 140), as mentioned Table 4. The alleles of Gt1 and Gt3 genotypes were found in 8 and 9 mixed samples, respectively, but no epidemiological link was found at the end between the corresponding patients. In addition, during the analysis of repeat samples from patients harboring multiple genotypes (Table 3), a change in the ratio of the alleles was observed (depicted as ***) in samples obtained 1 day apart (Patient7 and 21). Therefore, we decided not to determine genotype in samples harboring multiple alleles at more than one marker but only to search for and analyze already described genotypes.

The distribution of the alleles of each marker differed according to the patients’ underlying diseases. Notably, one allele of 5/6 markers of Gt21 were associated with renal transplant patients. The same analysis, after the removal of samples harboring Gt21, showed that one allele of only 2/6 markers were significantly associated with renal transplant patients.

We found Gt21 in 10 patients and built a putative transmission map. Other studies have demonstrated transmission within particular hospital environments (pediatric transplant unit, waiting room for renal transplant patients) [18,19,22]. Previous outbreaks have been suspected in our hospital [21,49], but this particular one was clinically unrecognized. However, the concomitant presence of some patients in the hospital on the same day prompted us to draw a transmission map suggesting possible transmission between these patients. Transmission could have occurred by meetings in various places (radiology room, cafeteria, corridors, hall) or through a third party (medical staff). Indeed, the possible role in transmission of an immunocompetent third-party such as carrier patients who did not develop PCP [16], family members or medical staff has already been suggested in other outbreaks [14,20,50].

Among the five genotype categories defined for the MSTree analysis, the major one, Cluster 1, included patients from all underlying disease groups and all geographical origins. Branches corresponding to patients with the same underlying disease (i.e. transplant recipients, hematological malignancy) were observed, suggesting an epidemiological link between the corresponding patients, which we failed to uncover. Cluster 2 was mostly composed of samples from renal transplant patients of various geographical origins. Based on the assumption that isolates from different origins exhibit different genotypes, our results confirm those of previous studies [2] suggesting that *P*. *jirovecii* does not reactivate the strain acquired during a primary infection, in contrast with cryptococcosis for example [51]. However, Parobek et al. have shown that *Pneumocystis* isolates were genetically close, despite having been recovered from highly distant geographical areas [44], suggesting a limited diversity of the alleles of their markers. The easiest way to demonstrate reactivation is to study the genotype of patients that migrated from their geographical origin to another country. However, if the allele diversity is low even considering various geographical places, typing methods based on STRs, in particular the assay published by Parobek et al., would not be suitable to investigate this way the reactivation hypothesis. In addition, BAL fluid samples would not be the best specimen to investigate reactivation since various genotypes could be recovered from various areas of the lungs. Induced sputa could better reflect the diversity of the genotypes that could be recovered from the whole lungs [52].

Singletons, i.e. samples with no genetic link with other genotypes recovered in the hospital were mostly composed of samples from HIV-positive patients from various geographical regions. PCP accounted for 32% of opportunistic infections in HIV-positive individuals in 2009 [53]; therefore, it is possible that these patients were not epidemiologically linked to the hospital before they were diagnosed with PCP. However, clinical criteria like (i) pneumocystosis, (ii) the number of episodes of PCP or (iii) previous visits as an outpatient before PCP, were not associated with the genotype categories or with specific alleles.

It is not possible to assess the discriminatory power of an assay with samples potentially involved in transmission, i.e. non-independent strains. Indeed, Simpson’s index of diversity (D) cannot be calculated with samples that are linked [43]. However, transmission is especially difficult to rule out with *P*. *jirovecii* strains within a hospital given the continual flux of chronically ill patients with a high risk of transmission. The genotype involved in the putative transmission in our hospital aggregated in cluster 2; therefore, we calculated the D index after the exclusion of samples from renal transplant recipients recovered from cluster 2. The resulting D index was then higher than that calculated with the whole population, but was still lower than that obtained by Parobek et al. (D≥0.999). This could be due to other unrecognized transmission events in our hospital, the limited diversity of *P*. *jirovecii* recovered from a relatively small area (Paris), or the use of six markers instead of nine. Alternatively, the diversity calculated by Parobek et al. may have been artificially high because of the potentially incorrect genotype assignment of samples harboring mixed alleles.

In conclusion, the assay described here is easy to perform. It should prove useful to investigate outbreaks in a hospital setting and should improve the understanding of the pathophysiology of *Pneumocystis* infections.

## Supporting Information

S1 File Intensity of the peaks for each marker tested for the internal control run nine times (Pj_SLS_181). (XLSX)

S2 File Allele of the 61 genotypes recovered in pure or mixed (more than one allele in one marker) samples. (XLSX)

S3 File Alleles detected for each marker for the 48 samples harboring more than one alleles in more than one locus. The 61 already determined genotypes were reported and counted in these samples.
(XLSX)

S1 Table Distribution of the different alleles of each marker in the different groups of diseases (DOCX)

## References

[1] Cushion MT (2010) Are members of the fungal genus *Pneumocystis* (a) commensals; (b) opportunists; (c) pathogens; or (d) all of the above? PLOS Pathog 6: e1001009 10.1371/journal.ppat.1001009 20885786 PMC2944789

[2] Gigliotti F, Wright TW (2012) *Pneumocystis*: where does it live? PLOS Pathog 8: e1003025 10.1371/journal.ppat.1003025.g001 23209406 PMC3510259

[3] Roblot F, Le Moal G, Godet C, Hutin P, Texereau M, Boyer E, et al (2003) *Pneumocystis carinii* pneumonia in patients with hematologic malignancies: a descriptive study. J Infect 47: 19–27. 10.1016/S0163-4453(03)00038-0 12850158

[4] Tasaka S, Tokuda H (2012) *Pneumocystis jirovecii* pneumonia in non-HIV-infected patients in the era of novel immunosuppressive therapies. J Infect Chemother 18: 793–806. 10.1007/s10156-012-0453-0 22864454

[5] Reid AB, Chen SCA, Worth LJ (2011) *Pneumocystis jirovecii* pneumonia in non-HIV-infected patients: new risks and diagnostic tools. Curr Opin Infect Dis 24: 534–544. Available: http://eutils.ncbi.nlm.nih.gov/entrez/eutils/elink.fcgi?dbfrom = pubmed&id = 21986616&retmode = ref&cmd = prlinks. 10.1097/QCO.0b013e32834cac17 21986616

[6] Wissmann G, Morilla R, Martín-Garrido I, Friaza V, Respaldiza N, Povedano J, et al (2010) *Pneumocystis jirovecii* colonization in patients treated with infliximab. Eur J Clin Invest 41: 343–348. 10.1111/j.1365-2362.2010.02415.x 21299548

[7] Catherinot E, Lanternier F, Bougnoux M-E, Lecuit M, Couderc L-J, Lortholary O (2010) *Pneumocystis jirovecii* pneumonia. Infect Dis Clin North Am 24: 107–138. 10.1016/j.idc.2009.10.010 20171548

[8] Mori S, Sugimoto M (2012) *Pneumocystis jirovecii* infection: an emerging threat to patients with rheumatoid arthritis. Rheumatology (Oxford) 51: 2120–2130. 10.1093/rheumatology/kes244 23001613 PMC3510430

[9] Pagano L, Fianchi L, Mele L, Girmenia C, Offidani M, Ricci P, et al (2002) *Pneumocystis carinii* pneumonia in patients with malignant haematological diseases: 10 years' experience of infection in GIMEMA centres. Br J Haematol 117: 379–386. 11972521 10.1046/j.1365-2141.2002.03419.x

[10] Chabé M, Dei-Cas E, Creusy C, Fleurisse L, Respaldiza N, Camus D et al (2004) Immunocompetent hosts as a reservoir of *Pneumocystis* organisms: histological and rt-PCR data demonstrate active replication. Eur J Clin Microbiol Infect Dis 23: 89–97. 10.1007/s10096-003-1092-2 14712369

[11] Gigliotti F, Harmsen AG, Wright TW (2003) Characterization of transmission of *Pneumocystis carinii* f. sp. *muris* through immunocompetent BALB/c mice. Infect Immun 71: 3852–3856. 12819069 10.1128/IAI.71.7.3852-3856.2003 PMC161994

[12] Choukri F, Menotti J, Sarfati C, Lucet JC, Nevez G, Garin JF, et al (2010) Quantification and spread of *Pneumocystis jirovecii* in the surrounding air of patients with *Pneumocystis* pneumonia. Clin Infect Dis 51: 259–265. 10.1086/653933 20572759

[13] Yazaki H, Goto N, Uchida K, Kobayashi T, Gatanaga H, Oka S, et al (2009) Outbreak of *Pneumocystis jiroveci* pneumonia in renal transplant recipients: *P*. *jiroveci* is contagious to the susceptible host. Transplantation 88: 380–385. 10.1097/TP.0b013e3181aed389 19667941

[14] de Boer MGJ, Bruijnesteijn van Coppenraet LES, Gaasbeek A, Berger SP, Gelinck LBS, van Houwelongen HC, et al (2007) An outbreak of *Pneumocystis jiroveci* pneumonia with 1 predominant genotype among renal transplant recipients: interhuman transmission or a common environmental source? Clin Infect Dis 44: 1143–1149. 10.1086/513198 17407029

[15] Pliquett RU, Asbe-Vollkopf A, Hauser PM, Presti LL, Hunfeld KP, Berger A, et al (2012) A *Pneumocystis jirovecii* pneumonia outbreak in a single kidney-transplant center: role of cytomegalovirus co-infection. Eur J Clin Microbiol Infect Dis 31: 2429–2437. 10.1007/s10096-012-1586-x 22402816

[16] Le Gal S, Damiani C, Rouillé A, Grall A, Tréguer L, Virmaux M, et al (2012) A cluster of *Pneumocystis* infections among renal transplant recipients: molecular evidence of colonized patients as potential infectious sources of *Pneumocystis jirovecii* . Clin Infect Dis 54: e62–e71. 10.1093/cid/cir996 22337822

[17] Olsson M, Eriksson BM, Elvin K, Strandberg M, Wahlgren M (2001) Genotypes of clustered cases of *Pneumocystis carinii* pneumonia. Scand J Infect Dis 33: 285–289. 11345221 10.1080/003655401300077324

[18] Gianella S, Haeberli L, Joos B, Ledergerber B, Wüthrich RP, Weber R, et al (2010) Molecular evidence of interhuman transmission in an outbreak of *Pneumocystis jirovecii* pneumonia among renal transplant recipients. Transplant infectious disease: an official journal of the Transplantation Society 12: 1–10. 10.1111/j.1399-3062.2009.00447.x 19744285

[19] Höcker B, Wendt C, Nahimana A, Tönshoff B, Hauser PM (2005) Molecular evidence of *Pneumocystis* transmission in pediatric transplant unit. Emerg Infect Dis 11: 330–332. 10.3201/eid1102.040820 15752458 PMC3320462

[20] Phipps LM, Chen SCA, Kable K, Halliday CL, Firacative C, Meyer W, et al (2011) Nosocomial *Pneumocystis jirovecii* pneumonia: lessons from a cluster in kidney transplant recipients. Transplantation 92: 1327–1334. 10.1097/TP.0b013e3182384b57 22129760

[21] de Castro N, Xu F, Porcher R, Pavie J, Molina JM, Peraldi MN, et al (2010) *Pneumocystis jirovecii* pneumonia in renal transplant recipients occurring after discontinuation of prophylaxis: a case–control study. Clin Microbiol Infect 16: 1375–1377. 10.1111/j.1469-0691.2009.03143.x 20041898

[22] Rabodonirina M, Vanhems P, Couray-Targe S, Gillibert R-P, Ganne C, Nizard N, et al (2004) Molecular evidence of interhuman transmission of *Pneumocystis* pneumonia among renal transplant recipients hospitalized with HIV-infected patients. Emerg Infect Dis 10: 1766–1773. 10.3201/eid1010.040453 15504262 PMC3323259

[23] Vargas SL, Hughes WT, Santolaya ME, Ulloa AV, Ponce CA, Cabrera CE, et al (2001) Search for primary infection by *Pneumocystis carinii* in a cohort of normal, healthy infants. Clin Infect Dis 32: 855–861. 10.1086/319340 11247708

[24] Schildgen V, Mai S, Khalfaoui S, Lusebrink J, Pieper M, Tillmann RL, et al (2014) *Pneumocystis jirovecii* can be productively cultured in differentiated cufi-8 airway cells. mBio 5: e01186–14–e01186–14. 10.1128/mBio.01186-14 24825015 PMC4030487

[25] Hauser PM, Blanc DS, Bille J, Telenti A, Francioli P (1996) Development of a molecular typing method for *Pneumocystis carinii* sp.f. hominis. J Euk Microbiol 43: 34S 8822837 10.1111/j.1550-7408.1996.tb04970.x

[26] Hauser PM (2004) The development of a typing method for an uncultivable microorganism: the example of *Pneumocystis jirovecii* . Infect Genet Evol 4: 199–203. 15450199 10.1016/j.meegid.2004.01.011

[27] Hauser PM, Francioli P, Bille J, Telenti A, Blanc DS (1997) Typing of *Pneumocystis carinii* f. sp. hominis by single-strand conformation polymorphism of four genomic regions. J Clin Microbiol 35: 3086–3091. 9399499 10.1128/jcm.35.12.3086-3091.1997 PMC230127

[28] Maitte C, Leterrier M, Le Pape P, Miegeville M, Morio F (2013) Multilocus sequence typing of *pneumocystis jirovecii* from clinical samples: how many and which loci should be used? J Clin Microbiol 51: 2843–2849. 10.1128/JCM.01073-13 23784120 PMC3754620

[29] Angulo B, García-García E, Martínez R, Suárez-Gauthier A, Conde E, Hidalgo M, et al (2010) A commercial real-time PCR kit provides greater sensitivity than direct sequencing to detect KRAS mutations: a morphology-based approach in colorectal carcinoma. J Mol Diagn 12: 292–299. 10.2353/jmoldx.2010.090139 20203003 PMC2860464

[30] Esteves F, Gaspar J, de Sousa B, Antunes F, Mansinho K, Matos O (2011) Clinical relevance of multiple single-nucleotide polymorphisms in *Pneumocystis jirovecii* pneumonia: development of a multiplex PCR-single-base-extension methodology. J Clin Microbiol 49: 1810–1815. 10.1128/JCM.02303-10 21389160 PMC3122690

[31] de Valk HA, Meis JFGM, Curfs IM, Muehlethaler K, Mouton JW, Klaassen CHW (2005) Use of a novel panel of nine short tandem repeats for exact and high-resolution fingerprinting of *Aspergillus fumigatus* isolates. J Clin Microbiol 43: 4112–4120. 10.1128/JCM.43.8.4112-4120.2005 16081958 PMC1233892

[32] Botterel F, Desterke C, Costa C, Bretagne S (2001) Analysis of microsatellite markers of *Candida albicans* used for rapid typing. J Clin Microbiol 39: 4076–4081. 10.1128/JCM.39.11.4076-4081.2001 11682532 PMC88489

[33] Enache-Angoulvant A, Bourget M, Brisse S, Stockman-Pannier C, Diancourt L, François N, et al (2010) Multilocus microsatellite markers for molecular typing of *Candida glabrata*: application to analysis of genetic relationships between bloodstream and digestive system isolates. J Clin Microbiol 48: 4028–4034. 10.1128/JCM.02140-09 20844221 PMC3020832

[34] Illnait-Zaragozi M-T, Martínez-Machín GF, Fernández-Andreu CM, Boekhout T, Meis JFGM, Klaassen CHW (2010) Microsatellite typing of clinical and environmental *Cryptococcus neoformans* var. *grubii* isolates from Cuba shows multiple genetic lineages. PLOS One 5: e9124 10.1371/journal.pone.0009124 20161737 PMC2817729

[35] Garcia-Hermoso D, Cabaret O, Lecellier G, Desnos-Ollivier M, Hoinard D, Raoux D, et al (2007) Comparison of microsatellite length polymorphism and multilocus sequence typing for DNA-based typing of *Candida albicans* . J Clin Microbiol 45: 3958–3963. 10.1128/JCM.01261-07 17928418 PMC2168554

[36] Garcia-Hermoso D, MacCallum DM, Lott TJ, Sampaio P, Serna M-JB, Grenouillet F, et al (2010) Multicenter collaborative study for standardization of *Candida albicans* genotyping using a polymorphic microsatellite marker. J Clin Microbiol 48: 2578–2581. 10.1128/JCM.00040-10 20427694 PMC2897530

[37] de Valk HA, Meis JFGM, Bretagne S, Costa JM, Lasker BA, Balajee SA, et al (2009) Interlaboratory reproducibility of a microsatellite-based typing assay for Aspergillus fumigatus through the use of allelic ladders: proof of concept. Clin Microbiol Infect 15: 180–187. 10.1111/j.1469-0691.2008.02656.x 19154486

[38] de Valk HA, Meis JFGM, Klaassen CHW (2007) Microsatellite based typing of *Aspergillus fumigatus*: strengths, pitfalls and solutions. J Microbiol Methods 69: 268–272. 10.1016/j.mimet.2007.01.009 17328980

[39] Cissé OH, Pagni M, Hauser PM (2012) De novo assembly of the *Pneumocystis jirovecii* genome from a single bronchoalveolar lavage fluid specimen from a patient. mBio 4 10.1128/mBio.00428-12 23269827 PMC3531804

[40] Benson G (1999) Tandem repeats finder: a program to analyze DNA sequences. Nucleic Acids Res 27: 573–580. 9862982 10.1093/nar/27.2.573 PMC148217

[41] Alanio A, Desoubeaux G, Sarfati C, Hamane S, Bergeron A, Azoulay E, et al (2011) Real-time PCR assay-based strategy for differentiation between active *Pneumocystis jirovecii* pneumonia and colonization in immunocompromised patients. Clin Microbiol Infect 17: 1531–1537. 10.1111/j.1469-0691.2010.03400.x 20946413

[42] Feil EJ (2004) Small change: keeping pace with microevolution. Nat Rev Micro 2: 483–495. 10.1038/nrmicro904 15152204

[43] Hunter PR, Gaston MA (1988) Numerical index of the discriminatory ability of typing systems: an application of Simpson's index of diversity. J Clin Microbiol 26: 2465–2466. 3069867 10.1128/jcm.26.11.2465-2466.1988 PMC266921

[44] Parobek CM, Jiang LY, Patel JC, Alvarez-Martinez MJ, Miro JM, Worodria W, et al (2014) Multilocus microsatellite genotyping array for investigation of genetic epidemiology of *Pneumocystis jirovecii* . J Clin Microbiol 52: 1391–1399. 10.1128/JCM.02531-13 24523468 PMC3993678

[45] Davidson S, Starkey A, MacKenzie A (2009) Evidence of uneven selective pressure on different subsets of the conserved human genome; implications for the significance of intronic and intergenic DNA. BMC Genomics 10: 614 10.1186/1471-2164-10-614 20015390 PMC2807880

[46] Nahimana A, Blanc DS, Francioli P, Bille J, Hauser PM (2000) Typing of *Pneumocystis carinii* f. sp. hominis by PCR-SSCP to indicate a high frequency of co-infections. J Med Microbiol 49: 753–758. 10933262 10.1099/0022-1317-49-8-753

[47] Esteves F, Gaspar J, Tavares A, Moser I, Antunes F, Mansinha K, et al (2010) Population structure of *Pneumocystis jirovecii* isolated from immunodeficiency virus-positive patients. Infect Genet Evol 10: 192–199. 10.1016/j.meegid.2009.12.007 20060502

[48] Hauser PM, Blanc DS, Sudre P, Senggen Manoloff E, Nahimana A, Bille J, et al (2001) Genetic diversity of *Pneumocystis carinii* in HIV-positive and-negative patients as revealed by PCR-SSCP typing. AIDS 15: 461–466. 11242142 10.1097/00002030-200103090-00004

[49] de Castro N, Neuville S, Sarfati C, Ribaud P, Derouin F, Gluckman E, et al (2005) Occurrence of *Pneumocystis jiroveci* pneumonia after allogeneic stem cell transplantation: a 6-year retrospective study. Bone Marrow Transplant 36: 879–883. 10.1038/sj.bmt.1705149 16151423

[50] Hauser P, Rabodonirina M, Nevez G (2009) Hypothetical *Pneumocystis jirovecii* transmission from immunocompetent carriers to infant. Emerg Infect Dis 15: 506–7–authorreply507. 10.3201/eid1503.081350 19239783 PMC2681130

[51] Garcia-Hermoso D, Janbon G, Dromer F (1999) Epidemiological evidence for dormant *Cryptococcus neoformans* infection. J Clin Microbiol 37: 3204–3209. 10488178 10.1128/jcm.37.10.3204-3209.1999 PMC85528

[52] Helweg-Larsen J, Lundgren B, Lundgren JD (2001) Heterogeneity and compartmentalization of *Pneumocystis carinii* f. sp. hominis genotypes in autopsy lungs. J Clin Microbiol 39: 3789–3792. 10.1128/JCM.39.10.3789-3792.2001 11574620 PMC88436

[53] de Castro N, Scemla A, Gallien S, Molina JM (2012) Pneumonie à *Pneumocystis jirovecii* chez les patients infectés par le VIH. Revue des maladies respiratoires 29: 793–802. 10.1016/j.rmr.2011.10.975 22742466

