# Supplementary material for: New Short Tandem Repeat-Based Molecular Typing Method for Pneumocystis jirovecii Reveals Intrahospital Transmission between Patients from Different Wards
Source: PLoS One. 2015 May 1;10(5):e0125763. doi: 10.1371/journal.pone.0125763 (PMC4416908; doi:10.1371/journal.pone.0125763)
Supplement: S1 Table — (DOCX) [file pone.0125763.s004.docx]

S1 Table : Distribution of the different alleles of each marker in the different groups of diseases

|  |  | number of samples in each group of disease harboring a given allele | | | |  |
| --- | --- | --- | --- | --- | --- | --- |
| Marker | Allele | HIV | Hematological  malignancies | Renal transplant | Other | p value |
| #022 | 138 | 37 | 23 | 1 | 10 | <0.0001 |
|  | 141 | 23 | 8 | 4 | 4 |  |
|  | 144 | 2*** | 4 | 10*** | 3 |  |
| #108 | 135 | 2 | 0 | 4 | 1 | 0.002 |
|  | 138 | 47 | 27 | 12* | 12 |  |
|  | 141 | 4 | 1 | 1 | 0 |  |
|  | 144 | 0 | 0 | 0 | 2 |  |
| #138 | 148 | 1 | 0 | 0 | 0 | 0.04 |
|  | 151 | 0 | 0 | 0 | 1 |  |
|  | 157 | 1 | 2 | 2 | 0 |  |
|  | 160 | 1 | 0 | 1 | 0 |  |
|  | 163 | 13 | 4 | 1 | 4 |  |
|  | 166 | 4 | 1 | 1 | 0 |  |
|  | 169 | 29 | 15 | 15** | 13 |  |
|  | 172 | 16 | 12 | 0 | 1 |  |
|  | 175 | 8 | 5 | 0 | 1 |  |
| #189 | 183 | 1 | 0 | 0 | 0 | 0.01 |
|  | 193 | 16 | 11 | 0 | 5 |  |
|  | 195 | 11 | 5 | 0 | 5 |  |
|  | 205 | 26 | 10 | 4 | 2 |  |
|  | 207 | 11 | 6 | 0 | 4 |  |
|  | 217 | 1 | 1 | 0 | 0 |  |
|  | 219 | 8 | 9 | 10*** | 6 |  |
|  | 221 | 0 | 2 | 0 | 2 |  |
|  | 229 | 0 | 0 | 1 | 0 |  |
| #278 | 187 | 4 | 1 | 0 | 0 | ns |
|  | 189 | 39 | 24 | 11 | 14 |  |
|  | 191 | 13 | 5 | 4 | 0 |  |
|  | 195 | 1 | 0 | 0 | 0 |  |
| #279 | 175 | 8 | 5 | 0 | 3 | <0.0001 |
|  | 178 | 24 | 13 | 0 | 6 |  |
|  | 181 | 31 | 15 | 4 | 5 |  |
|  | 184 | 11 | 2 | 1 | 0 |  |
|  | 187 | 2 | 1 | 0 | 2 |  |
|  | 190 | 3 | 3 | 10*** | 3 |  |

Alleles significantly associated with a group of disease are marked as *** (p<0.001), **(p<0.01), * (p<0.05)
